# Multidimensional Intersection of Nicotine, Gene Expression, and Behavior

**DOI:** 10.3389/fnbeh.2021.649129

**Published:** 2021-03-22

**Authors:** Yasmine Sherafat, Malia Bautista, Christie D. Fowler

**Affiliations:** Department of Neurobiology and Behavior, University of California, Irvine, Irvine, CA, Unites States

**Keywords:** nicotine addiction, nicotine withdrawal, nicotinic acetylcholine receptor (nAChR), gene expression, endogenous allosteric modulator, epigenetics, acetylcholine

## Abstract

The cholinergic system plays a crucial role in nervous system function with important effects on developmental processes, cognition, attention, motivation, reward, learning, and memory. Nicotine, the reinforcing component of tobacco and e-cigarettes, directly acts on the cholinergic system by targeting nicotinic acetylcholine receptors (nAChRs) in the brain. Activation of nAChRs leads to a multitude of immediate and long-lasting effects in specific cellular populations, thereby affecting the addictive properties of the drug. In addition to the direct actions of nicotine in binding to and opening nAChRs, the subsequent activation of circuits and downstream signaling cascades leads to a wide range of changes in gene expression, which can subsequently alter further behavioral expression. In this review, we provide an overview of the actions of nicotine that lead to changes in gene expression and further highlight evidence supporting how these changes can often be bidirectional, thereby inducing subsequent changes in behaviors associated with further drug intake.

## Introduction

The cholinergic system exerts widespread actions in multiple brain regions to regulate developmental processes, cognition, attention, motivation, reward, sleep, learning, and memory (Lindstrom, 1997; Picciotto et al., 2002; Blake and Boccia, 2018; Mu and Huang, 2019; Fowler et al., 2020; Gipson and Fowler, 2020). Deficits in cholinergic signaling are found to result in a broad range of negative impacts on cognitive processes, such as that found with Alzheimer’s disease, Parkinson’s disease, and other cognitive disorders (Lindstrom, 1997; Picciotto and Zoli, 2002). In the central nervous system, the endogenous neurotransmitter, acetylcholine, is released from axon terminals in all main subdivisions of the brain (Guo et al., 2015; Fowler et al., 2020). Acetylcholine acts on two main receptor subclasses, nicotinic acetylcholine receptors (nAChRs) and muscarinic acetylcholine receptors (mAChRs). The downstream effects of receptor activation not only modulate immediate behavioral and cognitive effects but also lead to downstream effects on gene expression.

Nicotine is an alkaloid derived from the tobacco plant and when consumed, acts as a full agonist on the nAChRs (Changeux et al., 1998; Picciotto et al., 2008). Tobacco cigarettes and e-cigarettes contain high levels of nicotine, which infer a high addiction liability through actions on nAChRs in the reward-related systems in the brain. Activation of nAChRs by nicotine leads to both similar and different actions as that found with acetylcholine, depending on the method of administration, dose, and frequency of exposure (McGehee et al., 1995; Picciotto et al., 2008; Chen et al., 2020; Fowler et al., 2020; Wittenberg et al., 2020). Moreover, in addition to changes in the brain, it should also be noted that long-term effects of nicotine are found in other organ systems, such as the lungs (Ariyoshi et al., 2002; Amos et al., 2008; Garcia-Arcos et al., 2016; Sun et al., 2017; Kyte and Gewirtz, 2018; Liu et al., 2018), although such peripheral actions of nicotine will not be reviewed further herein. In this review, we provide an overview of nicotine’s brain region and cell-type-specific actions on gene expression, and we further explore the bidirectional relationship between nicotine consumption and changes in gene expression.

## Nicotine’S Actions on Cholinergic Signaling Mechanisms

After release from the presynaptic terminal, acetylcholine binds to either mAChRs or nAChRs, depending on the receptor localization across cell types in the brain. Both receptors are involved in the autonomic nervous system, nociception, cognition, and learning and memory processes (Changeux et al., 1998; Yagi et al., 2015; Thomsen et al., 2018; Javadi-Paydar et al., 2019). The nAChRs are ionotropic, allowing for the influx of Na^2+^ and Ca^+^ and efflux of K^+^ during the receptor’s open conformational state following ligand binding (Lipsius, 1982; Fuentealba et al., 2004). In contrast, activation of the metabotropic mAChRs leads to G-protein mediated second-messenger signaling cascades (Haga, 2013). The mAChR exhibits five different subtypes (M1–M5; Hulme et al., 1990). M2 and M4 subtypes couple with Gi/Go proteins, whereas M1, M3, and M5 subtypes couple with Gq proteins (Haga et al., 2012). While nicotine acts directly on the nAChRs, but not the mAChRs, it appears that both receptor classes may modulate processes underlying drug addiction. For instance, a recent human GWAS study found that allelic variation in the M2 gene, *CHRM2*, is associated with nicotine dependence in women (Mobascher et al., 2010). Interestingly, activation of M2 mAChRs mediates aspects of pain perception, which is dysregulated in individuals with nicotine dependence (Wess et al., 2007; Al’Absi et al., 2015). These findings suggest a possible indirect link between nicotine, pain regulation, and mAChR signaling. Alternatively, nicotine-mediated changes in cholinergic levels (e.g., *via* nAChR localization on presynaptic terminals that modulate acetylcholine release) could impact endogenous volume transmission of acetylcholine within discrete brain areas, leading to altered activity at dendritic mAChRs (Yamasaki et al., 2010).

### Nicotinic Acetylcholine Receptors

In the brain, nAChRs are expressed on both neuronal and non-neuronal cells. These receptors are membrane-bound proteins from the Cys-loop family of the pentameric ligand-gated ion channels superfamily, which includes the serotonin 5-hydroxytryptamine type 3 receptor, l-aminobutyric acid type A receptor, and glycine receptor (Deba et al., 2018). As noted above, neuronal nAChRs can be expressed at either postsynaptic membranes or presynaptic terminals, and thus, nAChR activation can exert broad actions on multiple systems *via* modulation of neurotransmitter release from the synaptic terminal (Le Novère and Changeux, 1995; Mansvelder and McGehee, 2000; Deba et al., 2018). Heteromeric nAChRs are composed of a combination of α and β subunits, which can include α2-α7 and β2-β4, and α7 nAChR subunits can also combine to form a homomeric receptor (Le Novère and Changeux, 1995). The different subunits co-assemble to generate a wide variety of receptor subtypes with diverse patterns of expression across cell populations, and the specific combinations of α and β subunits can result in differences in ligand binding, Ca^2+^ permeability, and desensitization kinetics (Wang and Sun, 2005; Lipovsek et al., 2014; Pankratov and Lalo, 2014; Corradi and Bouzat, 2016; Zdenek et al., 2019). For example, in the ventral tegmental area (VTA), a variety of different nAChR subtypes have been proposed, which includes α4α5(β2)_2_, (α4)_2_(β2)_3_, (α4)_2_(β2)_3_, and α7 (Klink et al., 2001). Moreover, based on the specific localization within brain circuits, the effects of nAChR activation may lead to differing effects on behavior, which can be evidenced with nicotine self-administration; providing access across a range of doses leads to an inverted U-shaped dose-response curve that is indicative of both the reinforcing and aversive actions of the drug at the different doses (Fowler and Kenny, 2011; Fowler et al., 2011). Specifically, when nicotine doses increase from low to moderate, animals increase their responding to obtain more drug administrations, but in contrast, when doses increase from moderate to high, a decrease in responding is evidenced that is indicative of both aversion and satiation. Interestingly, the aversive properties of nicotine at higher doses are also evidenced by an increase in brain reward thresholds above baseline levels, as assessed with intracranial self-stimulation (Kenny, 2007; Fowler et al., 2011, 2013). The reinforcing and aversive properties of nicotine have been shown to involve opposing brain pathways, which are discussed in the following sections.

### Nicotine’s Actions on Brain Circuits Underlying Addiction

The reinforcing effects of nicotine are mediated by activation of the VTA, leading to increased dopaminergic signaling in the nucleus accumbens (NAcc; Picciotto, 1998; Rice and Cragg, 2004; Kenny and Markou, 2006; Mahler et al., 2019). The α4 and β2 nAChR subunits are expressed on the majority of dopaminergic and GABAergic neurons in the VTA and have been shown to modulate nicotine-evoked currents and reinforcing properties of the drug (Picciotto et al., 1998; Tapper et al., 2004; Changeux, 2010). For instance, knockout of α4, α6 or β2 nAChR subunits in mice results in a failure to self-administer nicotine, which can be rescued by re-expression of these nAChR subunits in the VTA (Picciotto et al., 1998; Pons et al., 2008). Given that the α4, α6 and β2 nAChRs can combine to form a functional nAChR subtype, it has been postulated that nicotine’s actions on the α4α6β2 nAChRs in the VTA-NAcc circuit control the reinforcing properties of the drug (Picciotto et al., 1998; Kenny and Markou, 2006; Pons et al., 2008; McGranahan et al., 2011). In contrast, the α7 nAChR subtype appears to modulate the fine-tuning of nicotine-mediated dopamine response with lower levels of expression on VTA neurons, as well as *via* localization on presynaptic glutamatergic terminals in the VTA (Jones and Wonnacott, 2004; Mameli-Engvall et al., 2006; Nashmi and Lester, 2006). Neurons in the VTA receive input from brain regions including the prefrontal cortex (PFC), amygdala, bed nucleus of the stria terminalis, tegmental nuclei, and local GABAergic interneurons (Theile et al., 2008; Mao et al., 2011), indicating multiple levels of modulation for integrative processing with the putative localization of nAChRs on axon terminals from some of these circuits. Cholinergic interneurons in the NAcc also play an important role in nicotine-seeking behavior (Leyrer-Jackson et al., 2020). Of note, chronic administration of nicotine enhances the functional connectivity between the cortex and NAcc during cue presentation with reward learning and has also been shown to increase NMDA signaling in the PFC-NAcc circuit (Ávila-Ruiz et al., 2014; Wang et al., 2020), thereby providing evidence of behavioral and circuit level changes in reward-related processing with nicotine use.

In opposition to the mesolimbic behavioral effects, the medial habenulo-interpeduncular (MHb-IPN) pathway plays an integral role in the aversive actions of nicotine and symptomology associated with nicotine withdrawal (Herkenham and Nauta, 1979; Salas et al., 2009; Fowler et al., 2011; Frahm et al., 2011; Fowler and Kenny, 2014; Antolin-Fontes et al., 2015; Pang et al., 2016; Sherafat et al., 2020). This pathway densely expresses several nicotinic subunits, including α3, α4, α5, β2 and β4 (Clarke et al., 1985, 1986; Mulle et al., 1991; Grady et al., 2009; Salas et al., 2009; Feduccia et al., 2012; Hendrickson et al., 2013). Of significance, in humans, polymorphisms of the *CHRNB4-CHRNA3-CHRNA5* gene cluster encoding for the α3, β4, and α5 nAChR subunits have been repeatedly associated with nicotine dependence (Freathy et al., 2009; Saccone et al., 2009; Zhang et al., 2010; Freathy et al., 2011). These nAChR subunits in the MHb-IPN pathway appear to modulate the aversive properties of nicotine, particularly at higher doses. Both constitutive knockout of the α5 nAChR subunit in mice and MHb-specific knockdown of the α5 nAChR gene in the rat leads to an increase in nicotine self-administration at higher doses (Fowler et al., 2011, 2013). In the knockout mice, α5 nAChR subunit re-expression in the MHb was sufficient to restore the behavioral phenotype, with no significant difference found in nicotine self-administration behavior compared to wildtype controls (Fowler et al., 2011). In contrast, mice with overexpression of the β4 nAChR gene exhibit a strong aversion to nicotine (Frahm et al., 2011). Finally, knockdown of the α3 nAChR subunit in the MHb or IPN increases nicotine intake at higher doses, a behavioral effect also found with the infusion of the α3β4 antagonist, a-conotoxin AuIB, into the IPN (Elayouby et al., 2021). Interestingly, the α3β4-containing nAChR has further been implicated in the physical signs of nicotine withdrawal (Jackson et al., 2013), which may involve altered GABAergic signaling in the IPN (Zhao-Shea et al., 2013). Finally, inhibition of these IPN GABAergic projections to the laterodorsal tegmentum has been shown to attenuate nicotine-conditioned place aversion (Wolfman et al., 2018). Therefore, given the multiple actions of nicotine on varying brain circuits, the downstream effects on gene expression can lead to a persistent impact on processes underlying nicotine use and abuse.

## Nicotinic Signaling and Gene Expression Along The Drug Use Trajectory

**Figure 1:**
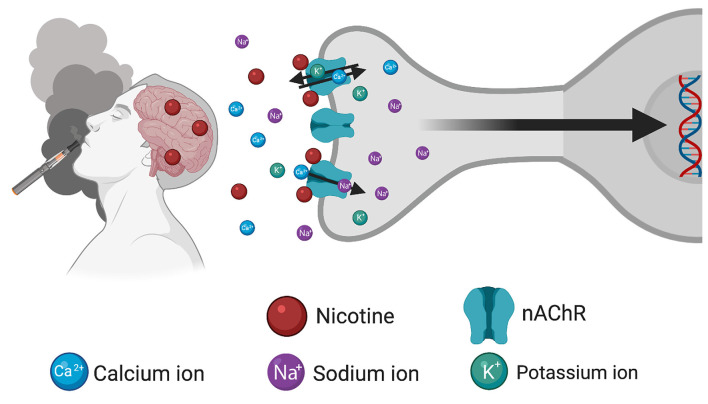
Schematic illustrating the consequences of inhalation of nicotine from tobacco/e-cigarette products to induce changes in gene expression. After entry into the brain, nicotine binds to and activates nicotinic acetylcholine receptors (nAChRs) located on the cellular membrane, thereby inducing an open conformational state permitting the influx of Ca^2+^ and Na^+^ ions and efflux of K^+^ ions. Subsequent changes in gene expression occur through the modulation of downstream signaling cascades. Created with BioRender.com.

Long-term changes in gene expression are thought to lead to behavioral habits and underlying processes that characterize the state of drug dependence, which can occur following consumption of nicotine-containing products including tobacco cigarettes and e-cigarettes/vapes (Figure 1). While the effects of nicotine on gene expression may be directly attributed to activation of nAChRs on the postsynaptic membrane, it is important to recognize that nAChRs can act as heteroreceptors to regulate the release of many neurotransmitters, in addition to acetylcholine, from the presynaptic terminal. Thus, nicotine’s effects on gene expression may be both directly on the nAChR-expressing cell, as well as indirectly with the involvement of multiple neurotransmitter systems. In the following paragraphs, we discuss evidence supporting interaction among nicotinic signaling, gene expression, and behavior, which may underlie various facets of drug use and abuse. These findings are further summarized in Table 1.

**Table 1:** Examples of gene expression changes with nicotine exposure.

Nicotine exposure	Direction of change	Brain region(s)	Genes	Functional implications	Citation(s)
Acute	Increase	AMG, Cortex, Hipp, IPN, MHb, NTS, SN, STR, VTA	*Fos*	Fos proto-oncogene; involved in neuronal activation; activation of these brain regions may be sufficient to alter nicotine intake.	Cole et al. (1989), Ren and Sagar (1992), Sharp et al. (1993), Picciotto et al. (1998), Tapper et al. (2004), Changeux (2010), Koob and Volkow (2010), Fowler et al. (2011, 2013), Frahm et al. (2011), Mineur et al. (2011), Picciotto and Mineur (2013), Zhao-Shea et al. (2013), Tuesta et al. (2017), Baur et al. (2018) and Dehkordi et al. (2018)
		Forebrain	*Arc*	Activity-regulated cytoskeleton-associated protein; involved in neuronal activation and plasticity; may increase reinforcing effects of nicotine.	Schochet et al. (2005) and Goriounova and Mansvelder (2012)
		PFC	*Ddn*	Dendrin; involved in learning and memory; adolescent-specific changes following acute nicotine.	Schochet et al. (2008), Doura et al. (2010) and Ji et al. (2019)
		VTA	*Gria1*	Glutamate ionotropic receptor AMPA subunit; involved in glutamatergic cell signaling; may enhance reinforcing response to nicotine.	Picciotto et al. (1998), Ferrari et al. (2002), Pons et al. (2008) and Yan et al. (2019)
		*In vitro (neuroblastoma cell line)*	*IL1B, IL6, CRELD2, PDIA6, HERPUD1*	Interleukin 1 beta and Interleukin; involved in the inflammatory response. Cysteine, rich with EGF like domains 2; may be involved in the transport of nAChRs. Protein disulfide isomerase family A member 6; involved in cell proliferation. Homocysteine inducible ER protein with ubiquitin-like domain 1; involved in stress response in the endoplasmic reticulum.	Ortiz et al. (2005), Hosur et al. (2009) and Bai et al. (2019)
	Decrease	IPN	*Pfn2*	Prolifin 2; involved in extracellular signaling.	Casserly et al. (2020)
Sub-Chronic	Increase	Hipp	*Crhr1, Crhr2*	Corticotropin-releasing hormone receptor 1 and 2; involved in the physiological stress response.	Carboni et al. (2018)
		Hipp, PFC, STR	*Crh*	Corticotropin-releasing hormone; involved in the physiological stress response.	Carboni et al. (2018)
		NAcc	*Creb1, Fosb*	CAMP responsive element binding protein 1; involved in the stimulation of the cAMP pathway. FosB proto-oncogene; involved in cell proliferation and differentiation; may increase cue-induced responses leading to increased nicotine seeking.	Zhu et al. (2007), Brunzell et al. (2009), Chen et al. (2014) and Alajaji et al. (2016)
		SNc	*Chrna6, Chrnb3*	α6 nAChR subunit and β3 nAChR subunit; involved in forming nAChRs on which nicotine directly binds.	Visanji et al. (2006) and Koob and Volkow (2010)
		VTA	*Oprd1*	Opioid receptor delta 1; involved in opioid dependence and aspects of nicotine action.	Ugur et al. (2017) and Fang et al. (2020)
	Decrease	Hipp, cortex	*Fos*	Fos proto-oncogene; involved in neuronal activation; the pattern of nicotine dosing indicates nAChR desensitization may contribute to conditioned drug reward.	Sharp et al. (1993) and Klink et al. (2001)
Chronic	Increase	AMG, OFC, mPFC, NAcc	*Fos*	Fos proto-oncogene; involved in neuronal activation; activation of these brain regions may be sufficient to alter nicotine intake.	Cole et al. (1989), Ren and Sagar (1992), Sharp et al. (1993), Picciotto et al. (1998), Tapper et al. (2004), Yu et al. (2008), Changeux (2010), Koob and Volkow (2010), Fowler et al. (2011, 2013), Frahm et al. (2011), Mineur et al. (2011), Picciotto and Mineur (2013), Zhao-Shea et al. (2013), Tuesta et al. (2017), Baur et al. (2018) and Dehkordi et al. (2018)
		Cortex, SN, VTA (GABAergic neurons)	*Chrna4*	α4 nAChR subunit; forms subtypes of nAChRs on which nicotine directly binds.	Picciotto (1998), Ferrari et al. (2002), Rice and Cragg (2004), Kenny and Markou (2006), Fasoli et al. (2016), Correa et al. (2019), Mahler et al. (2019) and Yan et al. (2019)
		Cortex, IPN (male-specific), IPN (female-specific during nicotine withdrawal), STR	*Chrb2*	β2 nAChR subunit; forms subtypes of nAChRs on which nicotine directly binds.	Staley et al. (2006), Fasoli et al. (2016) and Correa et al. (2019)
		Choroid plexus	*Mir204, Ttr*	MicroRNA 204; involved in regulating non-coding RNAs, anti-apoptotic signaling, linked to Schizophrenia in GWAS study. Transthyretin; involved in thyroid hormone and retinol transport, Aβ clearance in brain, linked to Alzheimer’s disease and Schizophrenia in GWAS study.	Serot et al. (1997), Wan et al. (2006), Turner et al. (2014b), Cammaerts et al. (2015) and Lallai et al. (2019)
		Hipp	*Nrg3, Creb1*	Neuregulin 3; involved in intracellular signaling, nicotine-related anxiety symptomology CAMP responsive element binding protein; involved in the stimulation of the cAMP pathway, nicotine-mediated responses, and withdrawal symptoms.	Turner et al. (2014a), Fisher et al. (2017) and Zhou et al. (2018)
		Hypothalamus	*Npy, Agrp*	Neuropeptide Y and Agouti-related neuropeptide; involved in food intake and weight regulation.	Huang et al. (2011)
		IPN	*Nos1, Sst*	Nitric oxide synthase 1 and Somatostatin; upregulation in α5 containing neurons contributes to signaling for nicotine withdrawal and aversion.	Herkenham and Nauta (1979), Salas et al. (2009), Fowler et al. (2011), Frahm et al. (2011), Fowler and Kenny (2014), Antolin-Fontes et al. (2015), Pang et al. (2016), Ables et al. (2017) and Sherafat et al. (2020)
		IPN (male-specific), SN, VTA	*Chrna7*	α7 nAChR subunit; forms homomeric subtype of nAChRs on which nicotine directly binds; involved in reward-related behaviors.	Panagis et al. (2000), Ryan and Loiacono (2001) and Visanji et al. (2006)
		IPN (female-specific)	*Chrna5*	α5 nAChR subunit; forms subtypes of nAChRs on which nicotine directly binds; contributes to nicotine withdrawal and aversion phenotypes.	Ables et al. (2017) and Correa et al. (2019)
		NAcc	*Nox2, Il1b, Tnf*	NADPH oxidase 2; involved in microglia morphology. Interleukin 1 beta; involved in the inflammatory response. Tumor necrosis factor; involved in cell proliferation.	Adeluyi et al. (2019) and Namba et al. (2020)
		PFC	*Mir199a, Mir214, Bdnf*	microRNA 199a and 214; implicated in cell proliferation with cancer. Brain-derived neurotrophic factor; involved in nerve growth, learning and memory, and cellular signaling.	Dhungel et al. (2018), Pittenger et al. (2018), Cole et al. (2020) and Tornesello et al. (2020)
		SN	*Chrna6, Chrnb3*	α6 nAChR subunit and β3 nAChR subunit; forms subtypes of nAChRs on which nicotine directly binds; correlated with increased nicotine self-administration.	Visanji et al. (2006), Quik et al. (2011) and Renda and Nashmi (2014)
		VTA	*Chrna5, Chrna6, Chrnb2, Dnm1, Ghr, Map*	α5 nAChR subunit, α6 nAChR subunit; β2 nAChR subunit; forms subtypes of nAChRs on which nicotine directly binds; may enhance reinforcing response to nicotine. Dynamin; involved in cellular membranes Growth hormone receptor; involved in cellular growth. Mitogen-Activated Protein; involved in cell proliferation.	Visanji et al. (2006), Doura et al. (2010), Berrendero et al. (2012) and Henderson et al. (2017)
	Decrease	Dorsal striatum	*Bdnf*	Brain-derived neurotrophic factor; involved in nerve growth, learning and memory, and cellular signaling.	Carboni et al. (2018)
		PFC	*Sirt1*	Sirtuin1; involved in epigenetic gene slicing.	Pittenger et al. (2018)
		PVN	*Crh*	Corticotrophin releasing hormone; involved in the physiological stress response.	Yu et al. (2008)
Prenatal	Increase	Whole-brain homogenization	*Il1B, Il6, Tlr4*	Interleukin 1 beta and Interleukin 6; involved in the inflammatory response. Toll-like receptor 4; involved in the immune response.	Chan et al. (2016)
		Hipp	*Slc6A4, Syne1*	Serotonin transporter; involved in serotoninergic signaling; may contribute to nicotine withdrawal symptoms. Spectrin repeat containing nuclear envelope protein 1; may contribute to nicotine withdrawal symptoms.	Mukhopadhyay et al. (2010) and Faulkner et al. (2018)
		PFC	CNTN4, EPHA8, GABRA4; Mobp*, Plp1*, Gje1* (*male-specific)	Contactin 4; involved in neuronal plasticity EPH receptor 8; involved in axonal projections. GABA-A receptor α4 subunit; involved in inhibitory signaling in the brain. Myelin associated oligodendrocyte basic protein and proteolipid protein; involved in forming the myelin surrounding nerve fibers. Gap junction protein epsilon 1; involved in ATP release.	Park et al. (1997), Bannon et al. (2005), Cao et al. (2013), Lubbers et al. (2014) and Semick et al. (2018)
		VTA	*Nurr1*	Nuclear receptor subfamily 4 Group A member 2; involved in dopaminergic signaling.	Romoli et al. (2019) and Rajan et al. (2020)
	Decrease	Forebrain, hindbrain	*Slc18A3, Slc5a7, Chat*	Vesicular acetylcholine transporter, High-affinity choline transporter, and choline acetyltransferase; involved in cholinergic signaling.	Mao et al. (2008)
		PFC	NRCAM; Mobp*, Plp1*, Gje1* (*female-specific)	Neuronal cell adhesion molecule; promotes directional axonal growth. Myelin associated oligodendrocyte basic protein and Proteolipid protein 1; both involved in forming the myelin surrounding nerve fibers. Gap junction protein epsilon 1; component of gap junctions for intercellular signaling.	Sonntag et al. (2009), Cao et al. (2013) and Semick et al. (2018)

*Acute, one injection; Sub-chronic, <10 injections/exposures; Chronic, >10 injections/exposures; AMG, amygdala; Hipp, hippocampus; IPN, interpeduncular nucleus; MHb, medial habenula; mPFC, medial prefrontal cortex; NAcc, nucleus accumbens; NTS, nucleus of the tractus solitarius; OFC, orbitofrontal cortex; PFC, prefrontal cortex; PVN, paraventricular nucleus; SN, substantia nigra; SNc, substantia nigra pars compacta; STR, striatum; VTA, ventral tegmental area*.

### Acute Nicotine Exposure

Experimenter-administered nicotine allows for precise dosing when one is interested in examining acute drug effects across experimental conditions, thereby elucidating findings particularly relevant to initial drug use. Acute nicotine exposure has been shown to increase the mRNA and protein expression of *c-fos*, an immediate early gene and marker of cellular activation, in various regions throughout the brain. For instance, nicotine-mediated increases in *c-fos* mRNA and/or protein expression have been documented in the VTA, MHb, and IPN, particularly at higher doses (Cole et al., 1989; Ren and Sagar, 1992; Sharp et al., 1993; Fowler et al., 2011; Baur et al., 2018). Since the VTA, MHb, and IPN have important roles in nicotine reinforcement and aversion, activation of these brain regions likely contributes to the drive to further consume the drug (Picciotto et al., 1998; Tapper et al., 2004; Changeux, 2010; Fowler et al., 2011; Frahm et al., 2011; Fowler et al., 2013). These activational effects are generally attributed to the direct actions of nicotine on the nAChRs, since pretreatment with the general nAChR antagonist, mecamylamine, can prevent such c-fos expression (Sharp et al., 1993). Given the regulatory regions in the *c-fos* gene, it is likely that the increased Ca^2+^ influx induced with nAChR opening leads to activation of second messenger systems, such as MAP- and CAM-kinases, thereby inducing the *c-fos* gene transcription (Kovács, 1998). In addition to reinforcement and aversion-related brain regions, nicotine-induced *c-fos* mRNA and/or protein expression has been evidenced in the hippocampus, substantia nigra, nucleus of the tractus solitarius, striatum, amygdala, and cortex, all of which appear to play a role in different aspects of drug dependence (Sharp et al., 1993; Koob and Volkow, 2010; Zhao-Shea et al., 2013; Tuesta et al., 2017; Dehkordi et al., 2018). Therefore, the extensive changes in *c-fos* gene expression provide insight into the broad actions of nicotine on neural signaling. Interestingly, repeated dosing appears to elicit nAChR desensitization in these actions, as a reduction in the expression of *c-fos* mRNA is found in the hippocampus and cortex after a second nicotine dose (Sharp et al., 1993); desensitization of nAChRs has been proposed to contribute to conditioned drug reward through salience of environmental cues associated with nicotine consumption (Klink et al., 2001). Moreover, while the c-fos protein has been shown to induce activation of multiple gene targets, one should note that: (1) expression may not change in cells exhibiting a net inhibitory state; (2) the protein may form a dimer with JunB to induce an inhibitory effect; and (3) the level of c-fos protein induced may not reach a sufficient threshold level to drive the transcriptional activity of all the target genes within a cell (Sheng and Greenberg, 1990; Kovács, 1998). Furthermore, while examining the acute actions of nicotine may provide some initial insights, chronic exposure conditions provide arguably more translational relevance for the changes in gene expression that likely occur in human smokers, which could alter neural function to potentially propagate the dependence state across time.

### Chronic Nicotine Exposure

Longer-term exposure to nicotine has been demonstrated to exert changes in gene expression across cell types and brain regions. In recently abstinent smokers, increased β2-containing nAChRs are found in the cortex and striatum (Staley et al., 2006). Data derived from animal models provides further evidence of increased nicotine-mediated expression for a subset of nAChR subunits in a region-specific manner (Marks et al., 1987, 1992; Adriani et al., 2003; Staley et al., 2006; Nashmi et al., 2007). For instance, chronic nicotine exposure leads to upregulation of the α6 and β3 nAChR subunits in the substantia nigra pars compacta (Visanji et al., 2006) and α7 nAChR subunits in the substantia nigra and VTA (Ryan and Loiacono, 2001). Also, the effects on nAChR expression involve differences in the assembly of the subunits to generate functional receptors at the membrane. Specifically, the α4 and β2 nAChR subunits can combine to form different isoforms with either two α4 subunits (α4_2_β2_3_) or three α4 subunits (α4_3_β2_2_). Chronic exposure to nicotine preferentially upregulates α4_2_β2_3_ nAChRs in the cortex, but not the thalamus (Fasoli et al., 2016), thereby demonstrating increased expression of the higher sensitivity α4_2_β2_3_ isoform following nicotine exposure in a region-specific manner. Additionally, the α4 and β2 subunits can also combine with the α5 subunit to form the α4_2_β2_2_α5 subtype, but the expression of this subtype does not change with nicotine exposure (Moretti et al., 2018). Further specificity is found for cell type specific patterns. Nicotine-mediated upregulation of α4-containing nAChRs occurs in the VTA and substantia nigra, but the increased expression is specific to GABAergic, not dopaminergic, neurons (Nashmi et al., 2007). With regard to the MHb-IPN circuit, α5 nAChR subunit expressing neurons in the IPN upregulate expression of the *Nos1* and *Sst* genes following repeated nicotine administration, leading to increased nitric oxide and somatostatin neurotransmitter release, respectively (Ables et al., 2017). Since blocking nitric oxide signaling also reduces nicotine preference (Ables et al., 2017), this further demonstrates a potential feedback mechanism for bidirectional effects between nicotine-seeking behavior and gene expression. Additionally, somatostatin has been shown to inhibit glutamate release in the IPN (Zhao-Shea et al., 2013), suggesting that upregulation of somatostatin could further serve to inhibit the activity of the aversion-related MHb-IPN circuit. Interestingly, sex differences have also been found in the IPN expression of acetylcholine and nAChRs following nicotine treatment. Specifically, females exhibit a greater increase in acetylcholine and α5 nAChR subunit mRNA levels, whereas males show an increase α7 and α2 nAChR subunit transcripts, in the IPN (Correa et al., 2019). It is also important to note that changes in nAChR subunit mRNA are not always associated with the level of protein expression at the membrane, and inversely, changes in nAChR membrane expression are not always a product of changes in nAChR gene expression. Indeed, membrane nAChR upregulation can be modulated by post-translational modifications or trafficking. For example, nicotine has been shown to upregulate receptors containing the α4 and β2 nAChR subunits *via* several mechanisms, including phosphorylation and chaperoning in the endoplasmic reticulum, which could lead to increased receptor insertion at the cell surface (Rothhut et al., 1996; Lester et al., 2009; Wecker et al., 2010; Srinivasan et al., 2011). Since α4 and β2 nAChR subunits in the VTA-NAcc circuit are necessary for nicotine reinforcement, the net effect of an upregulation in α4β2-containing nAChRs could be enhanced reinforcing effects of the drug at various doses (Picciotto et al., 1998; Tapper et al., 2004; Changeux, 2010).

Next, chronic nicotine exposure may lead to the modulation of membrane nAChRs through effects on other genes expressed in the cell. In a recent study, an innovative proteomics approach was taken to immunoprecipitate protein-protein interactions of β2-containing nAChRs and subsequently characterize the associated proteins (McClure-Begley et al., 2016). It was found that chronic nicotine exposure increases the expression of specific nAChR interacting proteins, including Na/K ATPases, syntaxins, SNAP25, and synaptotagmin, in the cortex of both mice and human smokers (McClure-Begley et al., 2016), thereby revealing the impact of nicotine on the expression of intracellular factors involved in nAChR regulation. In addition to nicotine, other constituents in tobacco and e-cigarette products may independently or synergistically act with nicotine to alter neuronal function. For instance, menthol is a common additive found both in tobacco cigarettes and e-cigarette vape solutions. When menthol and nicotine are co-administered, a significant upregulation of α4α6β2 nAChRs is found in the VTA, substantia nigra, and hypothalamus, as compared to nicotine alone (Henderson et al., 2017; Mulcahy et al., 2020), which is positively correlated with dopaminergic neuron excitability and increased drug reward (Henderson et al., 2017). Therefore, a bidirectional relationship is present between behavior and gene expression, in which drug consumption causes upregulation of nAChRs, which can subsequently affect neural responsiveness in brain regions that mediate aversion or reinforcement signaling that regulate later drug intake.

A variety of other genes have also been identified to be differentially regulated by nicotine exposure. In the VTA, increased mRNA expression of the delta-opioid receptor and GluA1 AMPA receptor are found with nicotine (Ferrari et al., 2002; Ugur et al., 2017; Fang et al., 2020); both of these receptors have been implicated in nicotine self-administration behavior (Kenny et al., 2009; Fowler et al., 2011; Berrendero et al., 2012). Further, decreased density of the metabotropic glutamate receptor 5 (mGluR5) has been documented in human smokers (Akkus et al., 2013). These findings were confirmed in rats following 250 days of nicotine exposure, in which decreased mGluR5 was found in the striatum, hippocampus, thalamus, and midbrain, and these changes in density were also associated with decreased exploratory behavior (Müller Herde et al., 2019). Abstinence following nicotine exposure restored mGluR5 expression (Müller Herde et al., 2019). However, another study examining nicotine-mediated changes in mGluR5 transcripts resulted in discrepant findings (Kane et al., 2005). Repeated nicotine injections have also been shown to induce expression of CREB in the NAcc, which appears to be essential for Pavlovian conditioning to nicotine-associated cues (Brunzell et al., 2009). Finally, nicotine can alter the expression of miRNAs in the brain, which may lead to subsequent effects on protein expression for multiple target genes. For instance, our recent study found that nicotine self-administration leads to an increase in mir204 and transthyretin in the choroid plexus of rats, both of which are released into the cerebrospinal fluid as circulating signaling factors (Cammaerts et al., 2015; Lallai et al., 2019; Sharma et al., 2019). Of note, both transthyretin and mir-204 have been implicated in regulating cell survival and may be involved in pathological states, such as Alzheimer’s disease and/or Schizophrenia (Serot et al., 1997; Wan et al., 2006; Turner et al., 2014b; Cammaerts et al., 2015; Li et al., 2016). Moreover, in females, but not males, nicotine self-administration was also found to induce an upregulation of mir199a and mir214 in the PFC, leading to a downregulation in protein expression of the target mRNA SIRT1 (Pittenger et al., 2018). Therefore, nicotine appears to induce actions on multiple genes across brain regions in a sex-dependent manner.

### Nicotine Withdrawal and Relapse

Nicotine withdrawal is characterized by several adverse symptoms in both human and rodent models, including increased anxiety and cognitive deficits (Damaj et al., 2003; West, 2006). Differential gene expression occurring during the withdrawal state may mediate symptomology. As noted above, the MHb-IPN circuit has been implicated in nicotine withdrawal. In the IPN, nicotine abstinence is associated with increased gene expression of the α4, α5, and β2 nAChR subunits (Correa et al., 2019) and decreased expression of the *Pfn2* gene (Casserly et al., 2020). Interestingly, knockdown of *Pfn2* in the IPN, but not in the VTA, results in an increased anxiety-related phenotype (Casserly et al., 2020), supporting a role in withdrawal-induced anxiety. The hippocampus has also been implicated in regulating anxiety-associated behaviors. During nicotine withdrawal, an increase in neuregulin 3 appears to mediate the synaptic plasticity that underlies anxiety-associated symptomology (Turner et al., 2014a; Zhou et al., 2018). Further, in the ventral hippocampus, the transcription factor CREB contributes to anxiety-associated behaviors during the withdrawal state (Fisher et al., 2017). Abstinence from drugs of abuse also activates stress-related signaling that involves corticotropin-releasing factor (CRF) and the associated CRF_1_ and CRF_2_ receptors (George et al., 2007). Following chronic nicotine, CRF levels are upregulated in the VTA, striatum, PFC, and hippocampus (Grieder et al., 2014; Zhao-Shea et al., 2015; Carboni et al., 2018), but downregulated in the paraventricular nucleus (Yu et al., 2008). Further, CRF_1_ and CRF_2_ receptors are found to be increased in the hippocampus (Carboni et al., 2018), and hippocampal CRF signaling has been implicated in anxiety-associated behaviors (Bertagna et al., 2021). Taken together, these findings demonstrate that a variety of gene expression changes during the withdrawal state may contribute to anxiety- and/or stress-related behavioral effects.

Deficits in cognitive function are also found with nicotine withdrawal. For instance, mice treated chronically with both moderate and high doses of nicotine exhibit impaired cognitive flexibility during a set-shifting task, and these deficits were associated with increased *Bdnf* gene expression in the medial PFC but decreased *Bdnf* in the dorsal striatum (Cole et al., 2020). Increased expression of nAChRs and CREB in the dorsal hippocampus has also been associated with learning deficits during nicotine withdrawal (Gould et al., 2014; Fisher et al., 2017), which also involves changes in other genes associated with long term potentiation for new contextual learning events, but not an expression of previously acquired contextual memory (Portugal and Gould, 2009). Finally, it is important to recognize the emerging importance of glial signaling in brain function. During nicotine withdrawal, proinflammatory effects have been associated with anxiogenic behaviors (Adeluyi et al., 2019). In the NAcc, nicotine withdrawal induces a change in microglial morphology, decreases astrocyte GFAP expression, and increases expression of Nox2, tumor necrosis factor-α, and interleukin-1β (Adeluyi et al., 2019; Namba et al., 2020). Thus, gene expression changes during abstinence from nicotine may contribute to various behavioral effects associated with the withdrawal state.

High relapse rates are often found when one attempts to quit the tobacco/nicotine smoking habit, and thus, ascertaining a better understanding of the neurobiological components modulating drug relapse may reveal new targets for therapeutic intervention. Drug relapse can involve the persistent memories of nicotine reward, aversive effects of withdrawal, and nicotine-associated conditioned stimuli (Herd et al., 2009; Pickens et al., 2011). Increased nicotine-seeking behavior after a prolonged period of abstinence is correlated with higher levels of c-fos protein expression in the amygdala, orbitofrontal cortex, medial prefrontal cortex, and NAcc (Funk et al., 2016). Further, glutamatergic plasticity in the NAcc, including alterations in the expression of the AMPA receptor and cholinergic interneuron function, has been shown to mediate cue-induced reinstatement (Vieyra-Reyes et al., 2008; Gipson et al., 2013; Leyrer-Jackson et al., 2020). Also, neuronal subpopulations within the basolateral amygdala appear to encode reward-associated memories, as selective inhibition of these neurons prevents nicotine seeking in both the conditioned place preference and incubation of craving paradigms (Xue et al., 2017). These findings support the notion that changes in gene expression may contribute to the likelihood of relapse-associated behaviors during abstinence.

Finally, weight gain following cessation may also contribute to high relapse rates, and studies examining the intersection of nicotine and feeding have found that nicotine induces c-fos protein expression in pro-opiomelanocortin and neuropeptide Y neurons of the arcuate nucleus, a brain region shown to mediate satiety (Fornari et al., 2007; Huang et al., 2011; Mineur et al., 2011; Picciotto and Mineur, 2013). Withdrawal from nicotine increases body weight, neuropeptide Y, and agouti-related protein expression in the hypothalamus, and these proteins have been implicated in regulating appetite and metabolism (Fornari et al., 2007). Together, these studies highlight a role for nicotine-related gene expression changes in body weight regulation, which may contribute to the propensity for drug use and relapse.

## Further Considerations for Nicotine-Mediated Changes in Gene Expression

Nicotine’s actions across the drug use trajectory may be modulated by several factors, in addition to nAChR receptor subtype expression and intracellular signaling cascades. In the following sections, we discuss additional considerations that can mediate nicotine’s actions at the various stages of drug use. First, we highlight the effects of nicotine across early developmental stages, and then we discuss the role of endogenous allosteric modulators that can play a key role in nicotine’s ability to induce a nAChR conformational change and/or modulate membrane expression (Morishita et al., 2010). Thereafter, we examine emerging evidence correlating changes in the epigenetic landscape with nicotine use.

### Developmental Effects of Nicotine on Gene Expression

While the above studies have focused on changes in gene expression occurring in the adult, it is important to consider the impact of nicotine during earlier developmental stages when the brain is highly susceptible to environmental influences. Maternal smoking has been associated with adverse consequences for the fetus, which includes altered development of cholinergic systems and low birth weight (Ernst et al., 2001). Importantly, the potential negative impact on the fetus is not restricted to high levels of smoking behavior, as second-hand smoke exposure has also been correlated with impaired cognitive development (Rauh et al., 2004). Studies of post-mortem tissue document altered gene expression in the prefrontal cortex of fetuses from smoking mothers, including increased expression of genes involved in neurodevelopment (*CNTN4, EPHA8*, and *GABRA4*) and decreased expression of genes involved in cell adhesion (*NRCAM*) and calcium signaling (*KCNN2*; Semick et al., 2018). However, for the most part, long-term behavioral outcomes in humans remain inconclusive due to potentially confounding factors, such as differential environmental, nutritional, and genetic influences among subjects. Thus, rodent models are essential to better elucidate the effects of developmental nicotine exposure on gene expression and behavior. A study from Mao et al. (2008) confirmed nicotine-induced dysregulation within the cholinergic system, in which early prenatal nicotine exposure led to decreased expression of choline acetyltransferase, vesicular acetylcholine transporter, and choline transporter in the forebrain and hindbrain of rats (Mao et al., 2008). Also, sidestream cigarette smoke exposure in dams, a model of second-hand exposure, resulted in dysregulation of numerous genes in the mouse hippocampus, including upregulated serotonin transporter gene *Slc6a4* and synaptic nuclear envelope gene *Syne1* (Mukhopadhyay et al., 2010). Importantly, sex-dependent changes have also been found with *in utero* nicotine exposure. The axon myelination-associated genes, *Mobp*, *Plp1*, and *Gje1*, were significantly upregulated in male, but downregulated in female, rat PFC following chronic gestational nicotine exposure (Cao et al., 2013). Maternal cigarette smoke exposure also resulted in increased expression of inflammatory signaling factors, including IL-1β, IL-6, and toll-like receptor 4 in adult male mice, although females were not examined in this study (Chan et al., 2016). Consistent with these findings, the impact of nicotine on immune signaling molecules has been further demonstrated with *in vitro* studies. In an α4 and β2 expressing neuroblastoma cell line, nicotine application downregulated gene expression of the inflammatory proteins IL-1β and IL-6 and increased expression of the endoplasmic reticulum proteins CRELD2, PDIA6, and HERPUD1 (Hosur et al., 2009). Interestingly, knockout of the CRELD2 protein increased α4 and β2 nAChR subunit expression, suggesting a bidirectional effect on gene regulation in this *in vitro* system (Hosur et al., 2009). Finally, a recent study has demonstrated that prenatal nicotine exposure increases the expression of *Nurr1* in the VTA, leading to a greater number of dopaminergic neurons and enhanced nicotine preference in adulthood (Romoli et al., 2019).

In addition to *in utero* developmental exposure, adolescence is a sensitive period for consequential effects of nicotine on the brain, which also involves sex-specific outcomes (Cross et al., 2017). Following nicotine exposure in adolescence, an upregulation in the expression of the nAChR subunits α5, α6, and β2, as well as genes associated with synaptic plasticity (*Dnm1*), neuron density (*Ghr*), and dendrite elongation (*Map*), is found in the VTA in adulthood (Adriani et al., 2003; Doura et al., 2010; Locker et al., 2016). Interestingly, many of these changes in gene expression are specific to nicotine exposure in adolescence, but not adulthood (Doura et al., 2010). Other brain regions, including the hippocampus, NAcc, and PFC, exhibit nicotine-mediated changes in gene expression following adolescent exposure, with differential effects based on the specific stage of adolescence (Polesskaya et al., 2007). Adolescent nicotine exposure also induces an upregulation in DeltaFosB in the NAcc (Alajaji et al., 2016), a transcription factor associated with neural changes underlying substance abuse (Nestler et al., 2001). Additional genes identified are involved in synaptic plasticity, including *Arc* and *Dendrin* (Schochet et al., 2005, 2008), suggesting that such long-term changes with adolescent nicotine exposure can mediate a variety of effects in adulthood underlying various affective, cognitive, and drug use behaviors (Goriounova and Mansvelder, 2012; Locker et al., 2016; Alajaji et al., 2016; Pushkin et al., 2019; Dukes et al., 2020). These studies demonstrate that nicotine consumption during adolescence can induce long-term changes in brain circuitry persisting into adulthood.

### Endogenous Modulators of Nicotinic Receptors

Recently, endogenous allosteric modulators have been found to associate with nAChRs to alter the ability of ligands, including nicotine or acetylcholine, to induce an open conformational state of the receptor (Delbart et al., 2018). By modulating nAChR activity at the membrane, the consequential significance of the allosteric modulator on gene expression can be both short- or long-lived *via* modulation of Ca^2+^ influx for subsequent second messenger signaling. In addition to effects on membrane-localized nAChRs, endogenous modulators may also affect the trafficking of nAChR subunits to the membrane *via* association in the endoplasmic reticulum (Nichols et al., 2014; Miwa et al., 2019). Moreover, different endogenous modulators have been found to preferentially interact with specific nAChR subtypes and to be expressed in a region- and cell-specific manner, allowing for a potential high specificity in their effects (George et al., 2017; Miwa et al., 2019; Anderson et al., 2020). For instance, RIC-3, the resistance inhibitors of cholinesterase 3, has been shown to increase α4 and β2 nAChR subunit protein expression, but not the assembly of these subunits into the α4β2 nAChR subtype (Dau et al., 2013). Whereas initial studies suggested that RIC-3 does alter assembly and cell surface trafficking of α7 nAChRs, but not α7 protein expression, it was subsequently demonstrated that RIC-3 can affect α7 nAChR expression but in a ratio-dependent manner (Wang et al., 2009; Dau et al., 2013; Ben-David et al., 2016). Specifically, RIC-3 decreases α7 nAChR expression, but *only* at low ratios of RIC-3 to α7 nAChR subunits (Wang et al., 2009; Ben-David et al., 2016). In addition to the independent actions of allosteric modulators, two different allosteric modulators may work together to exert effects on nAChRs. For example, NACHO is a small, multi-pass transmembrane protein enriched in the neuronal endoplasmic reticulum acts as a chaperone to mediate the assembly and surface expression of α7 nAChR subunits (Matta et al., 2017). When localized together, RIC-3 can interact with NACHO to differentially regulate the expression of the α7 nAChR subunit (Matta et al., 2017).

Prototoxins are another class of allosteric modulators, which are classified into the lymphocyte antigen-6 (Ly-6)/urokinase plasmogenic activating receptor (u-PAR) superfamily. Similar to RIC-3 and NACHO, prototoxins can associate with and differentially modulate nAChR expression and function through interactions in the endoplasmic reticulum, cytoplasm, and membrane surface (Wu et al., 2015). The identified protoxins include lynx1, lynx2, and lypd6, which can associate with nAChRs by anchoring adjacent to the receptor on the membrane *via* a GPI link (Dessaud et al., 2006; Holford et al., 2009; Tekinay et al., 2009). Lynx proteins act as a “molecular brake pad” of the cholinergic system, negatively modulating the nAChR to reduce its activity in the presence of an agonist (Kobayashi et al., 2014). Association of lynx proteins with nAChRs decreases nicotine- and acetylcholine-induced currents and increases desensitization (Miwa et al., 2006). Prototoxins can modulate cholinergic activity in a biologically applicable, spatially specific, and nAChR subtype-specific manner. For example, lynx1 has been shown to interact with the extracellular subunit interface of the nAChR, thereby altering receptor stoichiometry, nAChR assembly, and cell surface expression levels, leading to altered ligand-mediated cellular currents (Nichols et al., 2014). Lynx1 can alter the function of α6β2-, α4β2-, α3β4- and α7-containing nAChRs, as demonstrated in cell culture systems (Miwa et al., 2006; George et al., 2017). Lynx1 is highly expressed in brain regions implicated in nicotine dependence with localization in glutamatergic, GABAergic, and dopaminergic neurons (Sheffield et al., 2000; Demars and Morishita, 2014; Thomsen et al., 2014). Moreover, in dopaminergic neurons of the substantia nigra pars compacta, deletion of lynx1 reduces the function of the α6-containing nAChRs (Parker et al., 2017), indicating lynx1 modulation of α6-containing nAChRs occurs in these cellular populations. In contrast, lynx2 has been shown to interact with α4β2 nAChRs, to modulate nicotine’s effects on glutamatergic signaling in the prefrontal cortex, and to mitigate anxiety-related behaviors (Dessaud et al., 2006; Tekinay et al., 2009; Demars and Morishita, 2014; Wu et al., 2015). Thus, allosteric modulators can act in conjunction with nicotine (or acetylcholine) to modulate protein expression in a cell type- and brain region-specific manner, but further studies *in vivo* will be necessary to more clearly delineate the relative importance of this interaction on subsequent behavior related to nicotine dependence.

### Epigenetic Regulation

Epigenetic modifications can serve to increase or decrease gene promotor accessibility for transcriptional activation. Interestingly, increased methylation in the promoter region of the monoamine oxidase A gene, the enzyme that metabolizes serotonin and norepinephrine, has been linked with increased vulnerability for nicotine dependence in women (Philibert et al., 2008), and differences in DNA methylation can serve as a predictor of smoking behavior in humans (Corley et al., 2019). An elegant study in mice demonstrated that nicotine increases expression of the Ash2l/Mef2c complex during cortical development, which subsequently leads to changes in histone methylation in the promoter region of glutamatergic synaptic genes and representative changes in dendritic spine number and branching (Jung et al., 2016). In rats, chronic nicotine exposure has also been associated with a decrease in methylation of several genes in the medial prefrontal cortex, orbitofrontal cortex, and NAcc (Mychasiuk et al., 2013; Castino et al., 2018). In the prefrontal cortex, nicotine self-administration was correlated with decreased histone methylation at the H3K27me3 and H3K9me2 marks in the BDNF and cyclin-dependent kinase 5 gene, but in contrast, withdrawal from nicotine elicited a decrease in H3K14 acetylation at the BDNF promoter (Castino et al., 2018), demonstrating different changes across stages of drug use. Following nicotine abstinence, histone deacetylase inhibitor administration results in an attenuation of cue-induced reinstatement (Castino et al., 2015), suggesting a further role for epigenetic factors in relapse-associated behaviors. Thus, altered gene expression due to changes in the epigenetic landscape may contribute to the development and/or maintenance of nicotine dependence, withdrawal effects, or vulnerability to relapse.

Finally, it is worthwhile to note that *in utero* exposure to nicotine has also been associated with epigenetic changes. Because of the longevity of these changes, the effects of such regulation may lead to detrimental outcomes persisting through adolescence and into adulthood. For example, differential methylation patterns have been documented in blood samples following exposure to prenatal maternal smoking, with associations noted at CpG sites in the genes *MYO1G, FRMD4A, CYP1A1, CNTNAP2, ARL4C, AHRR, TIFAB, MDM4, AX748264, DRD1, FTO* (Richmond et al., 2018). Of note, some epigenetic changes associated with maternal tobacco exposure have been replicated in other studies in humans, can persist across the lifespan, and have been linked to schizophrenia-associated symptomology (Joubert et al., 2016; Richmond et al., 2018; Tehranifar et al., 2018; Wiklund et al., 2019). However, several caveats must be considered for human studies based on numerous potentially confounding factors, such as comorbidity of drug use, socioeconomic status, nutritional deficits, stress, etc., and as such, it is important to validate such findings in a more controlled model system. Further, epigenetic changes detected in blood samples may not relate to the processes occurring in the brain. Thus, additional studies have begun to validate and extend these findings with studies in rodents. For instance, nicotine administration to pregnant dams results in hypomethylated DNA in the fetal cortex (Chatterton et al., 2017), supporting the notion of brain-relevant epigenetic changes with nicotine exposure. Moreover, nicotine exposure in male mice, before copulation, leads to their offspring exhibiting methylation changes in hippocampal genes related to neural development and plasticity, which is correlated with increased fear conditioning and decreased nicotine reinforcement (Goldberg et al., 2021). Thus, these studies suggest that nicotine can induce epigenetic changes in early development through direct actions *in utero* in pregnant females, or by altering gene expression in male sperm before fertilization. However, future studies are necessary to more specifically delineate the causative effects of such changes in the epigenetic landscape.

## Conclusions

The impact of nicotine on cholinergic function and gene expression has been shown to modulate multiple downstream effects that can contribute to different facets of nicotine dependence. The relationship between nicotine-seeking behavior and gene expression is cell type-specific, sex-specific, developmental, and bidirectional. Moreover, nicotine’s actions on the cholinergic system can affect cognition, feeding, learning and memory, attention, and anxiety- and depression-associated symptoms, in addition to nicotine reinforcement and seeking behavior, and as such, the implications of nicotine use on gene expression is extensive and multifaceted (Changeux et al., 1998; Picciotto et al., 2002; Mineur et al., 2011; Koukouli et al., 2017). Thus, future efforts to more fully characterize the changes in gene expression occurring in specific cell types and brain regions at various stages along the drug use trajectory will be necessary to ascertain a comprehensive understanding of the intersectional and bidirectional contributions that regulate the relationship between gene expression and behavior. Through these analyses, novel gene targets may be identified as a foundation for more efficacious therapeutic development efforts.

## Author Contributions

All authors contributed to the article and approved the submitted version.

## Conflict of Interest

The authors declare that the research was conducted in the absence of any commercial or financial relationships that could be construed as a potential conflict of interest.
